# Multi-time resolution analysis of speech: evidence from psychophysics

**DOI:** 10.3389/fnins.2015.00214

**Published:** 2015-06-16

**Authors:** Maria Chait, Steven Greenberg, Takayuki Arai, Jonathan Z. Simon, David Poeppel

**Affiliations:** ^1^Neuroscience and Cognitive Science Program, University of MarylandCollege Park, MD, USA; ^2^Department of Linguistics, University of MarylandCollege Park, MD, USA; ^3^Silicon SpeechHidden Valley Lake, CA, USA; ^4^Department of Information and Communication Sciences, Sophia UniversityTokyo, Japan; ^5^Department of Biology, University of MarylandCollege Park, MD, USA; ^6^Department of Electrical and Computer Engineering, University of MarylandCollege Park, MD, USA; ^7^Institute for Systems Research, University of MarylandCollege Park, MD, USA; ^8^Department of Psychology, New York UniversityNew York, NY, USA; ^9^Department of Neuroscience, Max-Planck-InstituteFrankfurt, Germany

**Keywords:** speech perception, speech segmentation, temporal processing, modulation spectrum, auditory processing, syllable, phoneme

## Abstract

How speech signals are analyzed and represented remains a foundational challenge both for cognitive science and neuroscience. A growing body of research, employing various behavioral and neurobiological experimental techniques, now points to the perceptual relevance of both phoneme-sized (10–40 Hz modulation frequency) and syllable-sized (2–10 Hz modulation frequency) units in speech processing. However, it is not clear how information associated with such different time scales interacts in a manner relevant for speech perception. We report behavioral experiments on speech intelligibility employing a stimulus that allows us to investigate how distinct temporal modulations in speech are treated separately and whether they are combined. We created sentences in which the slow (~4 Hz; S_low_) and rapid (~33 Hz; S_high_) modulations—corresponding to ~250 and ~30 ms, the average duration of syllables and certain phonetic properties, respectively—were selectively extracted. Although S_low_ and S_high_ have low intelligibility when presented separately, dichotic presentation of S_high_ with S_low_ results in supra-additive performance, suggesting a synergistic relationship between low- and high-modulation frequencies. A second experiment desynchronized presentation of the S_low_ and S_high_ signals. Desynchronizing signals relative to one another had no impact on intelligibility when delays were less than ~45 ms. Longer delays resulted in a steep intelligibility decline, providing further evidence of integration or binding of information within restricted temporal windows. Our data suggest that human speech perception uses multi-time resolution processing. Signals are concurrently analyzed on at least two separate time scales, the intermediate representations of these analyses are integrated, and the resulting bound percept has significant consequences for speech intelligibility—a view compatible with recent insights from neuroscience implicating multi-timescale auditory processing.

## Introduction

A central issue in psycholinguistics, psychoacoustics, speech research, and auditory cognitive neuroscience concerns the range of cues essential for understanding spoken language and how they are extracted by the brain (Greenberg, 2005; Pardo and Remez, 2006; Cutler, 2012).

In the domains of psycholinguistics and speech perception, *phonetic segments or articulatory features* (e.g., Liberman and Mattingly, 1985; Stevens, 2002) and *syllables* (Dupoux, 1993; Greenberg and Arai, 2004) have been identified as fundamental speech units. A growing body of research, employing various experimental techniques, now points to the perceptual relevance of both feature- or segment-sized (estimates range from 25–80 ms) and syllable-sized (~250-ms) units in speech processing (see e.g., Stevens, 2002, for the role of features and Ghitza and Greenberg, 2009, for the role of syllables in decoding input). There remains, however, considerable controversy concerning the order in which these are extracted from the speech stream. More hierarchically inspired models, for example, assume that the analytic processes proceed strictly “left-to-right,” from smaller units [i.e., (sub-)phonemic information] to larger units (i.e., syllables), building larger representations in a feedforward, small-to-large manner (e.g., Gaskell and Marslen-Wilson, 2002; see Klatt, 1989, for an overview of such models).

Accumulating findings from the psychoacoustics literature are pointing to temporal modulations of similar sizes described above as the carriers of information critically relevant to speech intelligibility. Indeed, the temporal envelope of speech, which reflects amplitude modulation associated with articulator movement during speech production, has been a focus of intense investigation. These fluctuations in amplitude, at rates between 2 and 50 Hz, are thought to carry information related to phonetic-segment duration and identity, syllabification, and stress (Rosen, 1992; Greenberg, 2005). It is evident from various psychophysical studies under a range of listening conditions that the integrity of the temporal envelope is highly correlated with the ability to understand speech (Houtgast and Steeneken, 1985; Drullman et al., 1994a,b; Chi et al., 1999; Greenberg and Arai, 2004; Obleser et al., 2008; Elliott and Theunissen, 2009; Ghitza, 2012; Peelle et al., 2013; Doelling et al., 2014). A striking demonstration of listeners' ability to utilize such cues is provided by Shannon et al. (1995): excellent speech comprehension can be achieved by dividing the speech signal into as few as four frequency bands, extracting their temporal envelopes, and using these to modulate Gaussian noise of comparable bandwidth.

An influential study by Drullman et al. (1994a,b) investigated the effect of smearing the temporal envelope on intelligibility. They partitioned the speech spectrum (Dutch sentences and words) into narrow frequency bands and low-pass filtered (Drullman et al., 1994a) or high-pass filtered (Drullman et al., 1994b) the amplitude envelopes at different cutoff frequencies. The conclusion drawn from these studies is that most of the important linguistic information is in envelope components between 1 and 16 Hz, with a dominant component at around 4 Hz, corresponding to the average syllabic rate. Eliminating modulations at these frequencies blurs the boundaries between adjacent syllables; some studies have even suggested that only modulation frequencies below 8 Hz are truly relevant to intelligibility (e.g., Hermansky and Morgan, 1994; Kanedera et al., 1997; Arai et al., 1999). These findings are complemented by extensive recent functional brain imaging data showing that speech intelligibility is correlated with the ability of auditory cortical mechanisms to follow the frequency and phase of low-frequency modulations in the temporal envelope of the speech signal (Ahissar et al., 2001; Luo and Poeppel, 2007; Gross et al., 2013; Peelle et al., 2013; Ding and Simon, 2014; Doelling et al., 2014).

In many ways, the findings in speech psychoacoustics parallel conclusions from psycholinguistics. Temporal envelope fluctuations around 4-Hz coincide with the average duration of syllables and are generally thought to relate to syllabic-pattern information (Rosen, 1992; Greenberg, 1999, 2005; Ahissar et al., 2001; Ding et al., under review). The dependence of speech intelligibility on the integrity of these low modulation frequencies is consistent with studies describing the perceptual saliency of syllables in newborns and adults (Morais et al., 1979; Mehler et al., 1996). Higher temporal envelope frequencies are related to segmental information (Houtgast and Steeneken, 1985; Rosen, 1992; Shannon et al., 1995). Shannon et al. (1995) observed a decrement in speech perception performance when the temporal envelope was low-pass filtered at 16 Hz. This degradation affected recognition of consonants and sentences but not vowels. Moreover, neuropsychological studies show that speech and language disorders characterized by impaired segmental processing, such as dyslexia, are associated with a degradation of sensitivity to amplitude modulations in this range (Tallal et al., 1996; Rocheron et al., 2002; Witton et al., 2002; Lehongre et al., 2011). It is worth noting that alternative theories of dyslexia emphasize difficulties encoding the envelope, i.e., the longer, syllable-associated processing timescale (Goswami, 2011).

**Figure 1:**
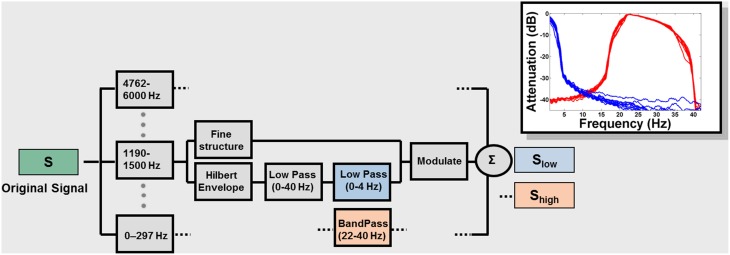
**Signal processing block diagram**. Signals were low-pass filtered at 6 kHz, sampled at 16 kHz, and quantized with 16-bit resolution. The frequency spectrum of the speech signal was partitioned into 14 frequency bands with a linear-phase FIR filter bank (slopes 60 dB/100 Hz or greater), spanning the range 0.1 and 6 kHz, spaced in 1/3 octave steps (approximately critical band-wide) across the acoustic spectrum. The Hilbert transform was used to decompose the signal in each band into a slowly varying temporal envelope and a rapidly varying fine structure. The temporal envelope was subsequently low-pass filtered with a cutoff frequency of 40 Hz and then either low- (0–4 Hz; blue blocks) or band- (22–40 Hz) pass filtered (red blocks). The time delays, relative to the original signal, introduced by the filtering, were compensated by shifting the filter outputs. After the filtering, the envelope was combined with the carrier signal (fine structure) by multiplying the original band by the ratio between the filtered and original envelopes. The result for each original signal (S) is S_low_ and S_high_, containing only low or high modulation frequencies. The inset shows the effect of the signal processing on a sample sentence. The attenuation caused by the filtering is plotted as a function of the frequency content of the envelopes. Blue: envelopes (for each of the 14 frequency bands) of S_low_; Red: envelopes of S_high_.

Notwithstanding the considerable evidence for feature and segmental analysis, on the one hand, and syllabic processing on the other, it is not understood whether information associated with the different modulation frequencies (and time scales) interacts in a manner relevant for speech perception. Here we describe a method for systematically probing the extraction and combination of these putative informational constituents of speech. We employed a modulation spectral processing scheme similar to Drullman et al. (1994a,b). Using this technique, we created sentences in which the slow (~4 Hz; S_low_) and rapid (~33 Hz; S_high_) modulations (corresponding to ~250 and ~30 ms, the average durations of syllables and certain phonetic properties, respectively) were selectively extracted in order to determine how intelligibility depends on information associated with these different time scales (Figure 1). In Experiment 1, we compared the performance of listeners presented with sentences containing low-frequency modulations alone (S_low_; <4 Hz; “LOW”), high-frequency modulations alone (S_high_; 22–40 Hz; “HIGH”), or a dichotic presentation of both types of information (S_low_ and S_high_; “BOTH”). We demonstrate that presentation of S_low_ with S_high_ results in significantly better intelligibility compared to the presentation of each signal separately. Such data imply an interactive binding process by which a conjunction of low- and high-modulation frequency information creates an integrated representation that is more useful (supra-additive performance) for speech recognition than a mere linear combination would imply. In Experiment 2, we investigate one temporal parameter governing this binding process by delaying one signal relative to the other and examining the impact of this stimulus onset asynchrony on intelligibility. Together, these experiments provide some of the first psychophysical evidence for the interaction of the two time scales during the decoding of spoken language.

## Materials and methods

### Experiment 1

#### Subjects

Thirty three subjects (19 female), between 18 and 41 (mean 22.5 years), took part in Experiment 1. All were native speakers of American English, right handed, and reported normal hearing as well as no history of neurological disorder. The experimental procedures were approved by the University of Maryland Institutional Review Board, and written informed consent was obtained from each participant. Subjects were paid for their participation or received course credit.

#### Stimuli and signal processing

Figure 1 describes the signal-processing technique used (see also Silipo et al., 1999) which is an extension of the method used in Drullman et al. (1994a,b). The result for each original signal (S) is S_low_ and S_high_, containing only low or high modulation frequencies (Figure 1, inset). Filter parameters were chosen to encompass the modulation frequencies shown to be most relevant for speech: 4 Hz (~250-ms-sized temporal windows) in the S_low_ condition and 33 Hz (~30 ms temporal windows) in the S_high_ condition. These values are further motivated by the pervasive relevance of these time intervals in non-speech and brain-imaging studies (see Zatorre and Belin, 2001; Poeppel, 2003; Boemio et al., 2005; Hesling et al., 2005; Giraud et al., 2007; Telkemeyer et al., 2009 and references therein; Giraud and Poeppel, 2012; Luo and Poeppel, 2012; Saoud et al., 2012). In order to study the interaction between the different types of information, we chose to separate S_low_ and S_high_ as much as possible in the modulation-frequency domain (Figure 1). This separation comes at the cost of significant information reduction in the signal and consequently a decline in intelligibility (see discussion below).

The original speech signals were 53 meaningful, syntactically varied, low-context sentences from the “Harvard phonetically-balanced sentences” corpus read by a female American English speaker (IEEE, 1969; Rabinowitz et al., 1992). Additional sentences were used for practice. The length of each sentence was ~2.5 s. The average number of words in a sentence was 7.8 (min = 5; max = 10).

There were three experimental conditions for each sentence: S_low_ presented diotically (same signal played to the two ears; LOW), S_high_ presented diotically (HIGH) and S_low_ and S_high_ presented dichotically (one to each ear; BOTH). We presented S_low_ and S_high_ dichotically, rather than combining them into a monaural presentation, in order to force the auditory integration to occur as far upstream along the neuraxis as possible. Both types of information are normally available in the input to each ear, but to investigate the extraction of the low- and high-modulation-frequency information from S_low_ and S_high_, respectively, we sought to eliminate interactions in the auditory periphery, such that any change in performance associated with the presentation of S_low_ and S_high_ concurrently would not be the result of acoustic fusion *per se*, but rather reflect a more abstract level of processing (e.g., phonetic features) (Cutting, 1976).

Word and syllable report scores from spoken sentences are influenced by a variety of factors, ranging from high-level sentential context to low-level acoustic properties. The use of this measure in assessing acoustic/phonetic processing of speech therefore requires careful control over other, bottom-up influences on report scores. Stimuli (53 sentences × three presentation conditions) were divided into three lists, each containing all 53 sentences and an equal number of trials of each presentation condition type. Each list contained only one presentation condition (LOW, HIGH or BOTH) per sentence (i.e., no repetition of sentences within list), minimizing top-down effects on intelligibility performance. Ten additional sentences, which were used in only a single condition (four BOTH, three LOW and three HIGH), were included in the experiment. These items were identical across all lists, and were used to compare subject performance. They are included in the analysis by subjects but not in the analysis by items. Subjects were randomly assigned to each list, and the order of presentation of sentences within each list was randomized. The ear of presentation in the BOTH condition was counter-balanced such that half of the subjects heard the first half of the stimuli with LOW in left ear and HIGH in right ear (or vice versa). For the other half, this order was reversed. We chose not to have the ear of presentation completely randomized because of concerns this could introduce an additional burden for the subjects (given that the task was difficult and required adaptation to the stimuli). For the same reason, we chose to present the LOW and HIGH conditions binaurally and not monaurally.

The stimuli were created off-line, saved *in stereo* WAV format at a sample rate of 16 kHz, and presented to participants by custom-made stimulus delivery software on a PC computer. The stimuli were played over high-quality headphones (Sennheiser HD580) at a comfortable listening level (under subject control; between 60 and 70 dB SPL) in a quiet room.

#### Procedure

The experiment lasted ~1 h. Subjects were instructed to type as many words of each sentence as possible and encouraged to guess when uncertain. Each stimulus was presented three times. Participants controlled when the stimuli were played by pressing a “play” button and were allowed to type their response at any time. After the third presentation, the subject had to complete his/her response and press a different button to initiate the next trial.

Each listener was presented with 26 practice sentences (played in the same order to all subjects) before beginning the experiment proper. The practice sentences contained exemplars of all three experimental conditions (LOW, HIGH and BOTH) as well as “clear” (i.e., unprocessed) sentences. In the practice session, some sentences were presented more than once (for example, an unprocessed and then a processed version of the same sentence, or vice versa) in order to facilitate learning (Davis et al., 2005). No feedback was provided in either the practice or test sessions.

### Data analysis

Each sentence in the IEEE Harvard sentence corpus contains five pre-marked key words. The intelligibility scores were computed in several ways: (i) by scoring the number of keywords correct out of the total number of key words in a sentence; (ii) the number of syllables correct in keywords out of the total number of syllables in keywords; and (iii) the number of syllables correct in all words out of the total number of syllables. All methods yielded qualitatively similar results. For this reason, we report only the intelligibility scores derived from counting the number of syllables correct in all words (iii). Function words such as “the,” “a,” and “an,” were not scored. Responses to a word received half credit if its morphology was incorrect (e.g., *hat* instead of *hats* or *danger* instead of *dangerous*) but otherwise semantically appropriate. Full credit was given for homonyms (e.g., “*read*” instead of “*red*”). Practice sentences were not scored.

Due to a software logging error, information associated with ear of presentation was lost for Experiment 1; therefore the effect of ear of presentation could not be analyzed. However, such information was analyzed in Experiment 2. Statistical tests were assessed with two-tailed tests, the α level was set *a-priori* to 0.05.

### Experiment 2

#### Subjects

One hundred sixty six subjects (113 female), between the ages of 18 and 53 (mean 21 years), took part in Experiment 2. All were native speakers of American English, right handed, reported normal hearing and no history of neurological disorder. The experimental procedures were approved by the University of Maryland Institutional Review Board, and written informed consent was obtained from each participant. Subjects were paid for their participation or received course credit.

#### Stimuli and signal processing

Due to a limitation on experiment duration, and the need for a longer practice session in Experiment 2, we selected a subset of 36 sentences from the set used in Experiment 1. In order to produce a sufficiently large dynamic range with which to measure the impact of asynchrony on intelligibility, we selected sentences in which the performance on the HIGH and LOW conditions alone was relatively poor but where their conjunction resulted in significantly higher performance. The average scores for the selected sentences were 18% in the HIGH condition, 41% in the LOW condition, 70% in the BOTH condition, and 50% for the PREDICTED variable (see below).

All of the stimuli used in Experiment 2 were dichotic, with S_low_ played to one ear and S_high_ to the other. They were generated using the same process as described in Experiment 1, except that we introduced a delay of S_low_ relative to S_high_. The onset delays were incremented in 15-ms steps between 0 and 105 ms and in ca. 50-ms steps between 105 and 350 ms, resulting in 13 delay conditions (0, 15, 30, 45, 60, 75, 90, 105, 150, 200, 250, 300, and 350 ms). Another manipulation concerned whether S_low_ was leading or trailing S_high_, resulting in a total of 25 experimental conditions. The stimuli were divided into 25 lists, each containing all 36 sentences and all 25 conditions, but only one condition per sentence. Subjects were randomly assigned to each list, and the order of sentence presentation within each list was randomized.

The ear of presentation in the BOTH condition was counter-balanced such that half of the subjects heard the first half of the sentences with the LOW signal in the left ear and the HIGH signal in the right ear. For the other half, this order was reversed. We chose not to have the ear of presentation completely randomized because of concerns that complete randomization of conditions would complicate the listening task beyond the requirements of the study.

#### Procedure

The procedure was the same as in Experiment 1. The experimental run lasted ~1 h. Each participant listened to 40 practice sentences (presented in the same order to all subjects) before beginning the experiment proper. The practice sentences contained several representative experimental conditions (asynchrony values of 0, 30, 75, 100, 200, and 300 ms), as well as clear (un-processed) sentences. The practice session also contained a small number of LOW and HIGH condition sentences (although these conditions did not appear in the experiment proper). The purpose was to focus the subjects' attention on the different types of information played to the two ears.

#### Data analysis

As for Experiment 1, we report intelligibility scores derived from scoring the number of syllables correct in all words. Practice items were not scored. Experiment 2 was much more difficult for listeners than Experiment 1. Because of the large number of experimental conditions and the relatively short practice period, subjects heard fewer “easy” (synchronous or small-delay stimuli) sentences. We observed significant learning effects such that average performance on the last half of the sentences presented was significantly better than on the first half. Limitations of time and the need to maintain subjects' attention and vigilance precluded lengthening the practice session, but we achieved a comparable effect by including in the analysis only the final half of the material for each subject (the large number of subjects, and the between-subjects design allow for this manipulation).

## Results

### Experiment 1

**Figure 2:**
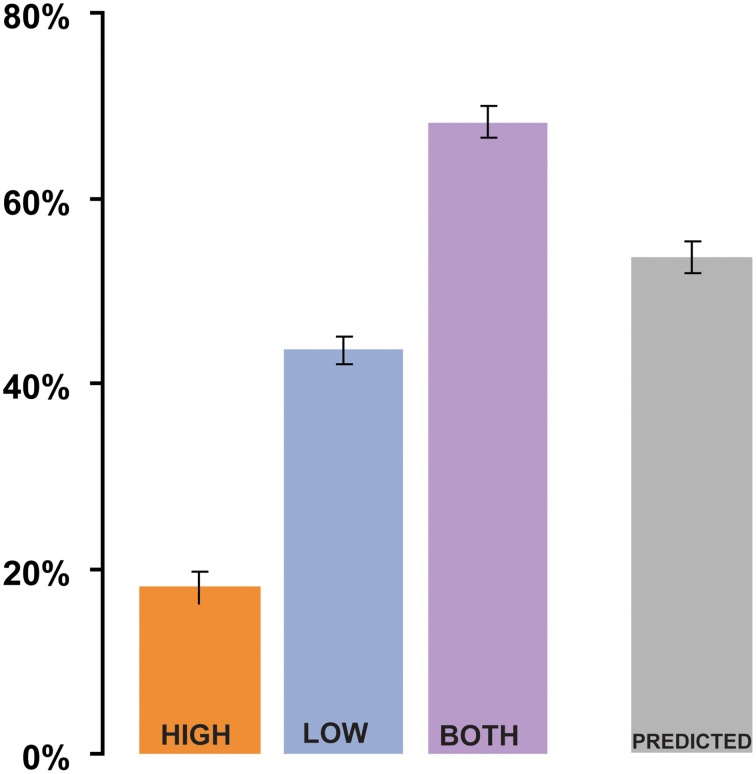
**Results of Experiment 1 (analysis by subjects)**. S_high_ and S_low_, when presented separately (HIGH and LOW conditions, respectively), have low intelligibility. Dichotic presentation of S_high_ with S_low_ (BOTH) results in significantly better performance than what could be predicted from the combined performance on the HIGH and LOW conditions (“Linear combination”). Error bars are 1 std. error.

The results of Experiment 1 are summarized in Figure 2. In the analysis by subjects, the mean intelligibility score was 17% in the HIGH condition, 42% in the LOW condition, and 66% in the BOTH condition, with similar results in the analysis by items (19% in HIGH, 39% in LOW, and 64% in BOTH). Intelligibility in the HIGH condition was not as good as that reported by Drullman et al. (1994b), even though we used similar signal-processing methods. The differences are probably attributable to the low transition probability of the words contained in the sentential material used in our study. Good intelligibility in the LOW condition is consistent with previous findings regarding the importance of low-frequency modulations to speech comprehension (Drullman et al., 1994a; Hermansky and Morgan, 1994; Ahissar et al., 2001; Greenberg and Arai, 2004; Elliott and Theunissen, 2009; Ghitza and Greenberg, 2009). As we were interested in examining the relative contribution of the different types of information, it was necessary to filter the original acoustic signals in a way that would maintain a high degree of separation in modulation-frequency space between the LOW and HIGH conditions (see inset in Figure 1). This separation comes at a cost of a significant reduction of information in the signal and a decline in overall intelligibility. Nevertheless, the values for the BOTH condition are similar to intelligibility reports over the same sentential material for unfiltered-noise-modulated envelopes (Zeng et al., 2005). Crucially, intelligibility is significantly increased (relative to LOW conditions) when both low and high frequency modulation information is available to the listener [repeated measures ANOVA: by subjects: *F*_(1, 35)_ = 222.4 *p* < 0.0001; analysis by items: *F*_(1, 52)_ = 209, *p* < 0.0001]. This finding is inconsistent with claims that only low-frequency modulations contribute to speech understanding.

Subjects often reported that information in the ear receiving the high modulation frequency information was completely “noisy” and they were trying to ignore it. Notwithstanding listeners' subjective reports, the analysis shows that the addition of the HIGH condition to the LOW condition significantly *improved* performance—“binding” of information carried in the two modulation-frequency bands apparently occurred despite subjects' attempts to ignore the high modulation-frequency signal.

To evaluate the relationship between the performance on HIGH and LOW compared to the BOTH condition, we created a derived variable PREDICTED: the value predicted from the combined performance on the HIGH and LOW conditions. This variable was computed for each subject by using the equation: PREDICTED = 1 − (*E*_low_ × *E*_high_), where *E*_low_ = 1 − LOW and *E*_high_ = 1 − HIGH are the error rates associated with the LOW stimuli and HIGH stimuli (the proportion of syllables incorrectly identified; Blamey et al., 1989). The predicted variable is based on the (overly conservative) assumption that the S_low_ and S_high_ signals independently contributed to intelligibility, and that an error occurred in the combined presentation only if a word was incorrectly perceived in *both* LOW and HIGH. The PREDICTED value (see Figure 2; 52% in the analysis by-subjects, 49% in the analysis by-items) was compared to the BOTH condition using a repeated-measures ANOVA. The comparison shows that performance on the BOTH condition was significantly better [by-subjects analysis: *F*_(1, 35)_ = 92, *p* < 0.0001; by-items analysis: *F*_(1, 52)_ = 58.3, *p* < 0.0001] than would be expected from integration of independent information from HIGH and LOW signals.

The PREDICTED variable is an *upper limit* on the performance that can be expected were subjects solving the task by linearly combining the information from HIGH and LOW. For example, if performance on LOW and HIGH is correlated, which is indeed the case (items analysis shows a Pearson's *r* = 0.529 *p* < 0.0001), a linear combination will result in a value that is *lower* than PREDICTED. The only situation in which linear combination performance might be expected to surpass PREDICTED is if LOW and HIGH were anti-correlated (no overlap between the words reported), which is ruled out by the positive correlation above. Consequently, the significantly better performance on BOTH relative to PREDICTED (an increase of *at least* 15%) suggests a non-linear interaction between the performance on LOW/HIGH and BOTH—indicative of a binding process in which the two information streams are combined to create a composite representation that is more than the sum of its parts.

### Experiment 2

**Figure 3:**
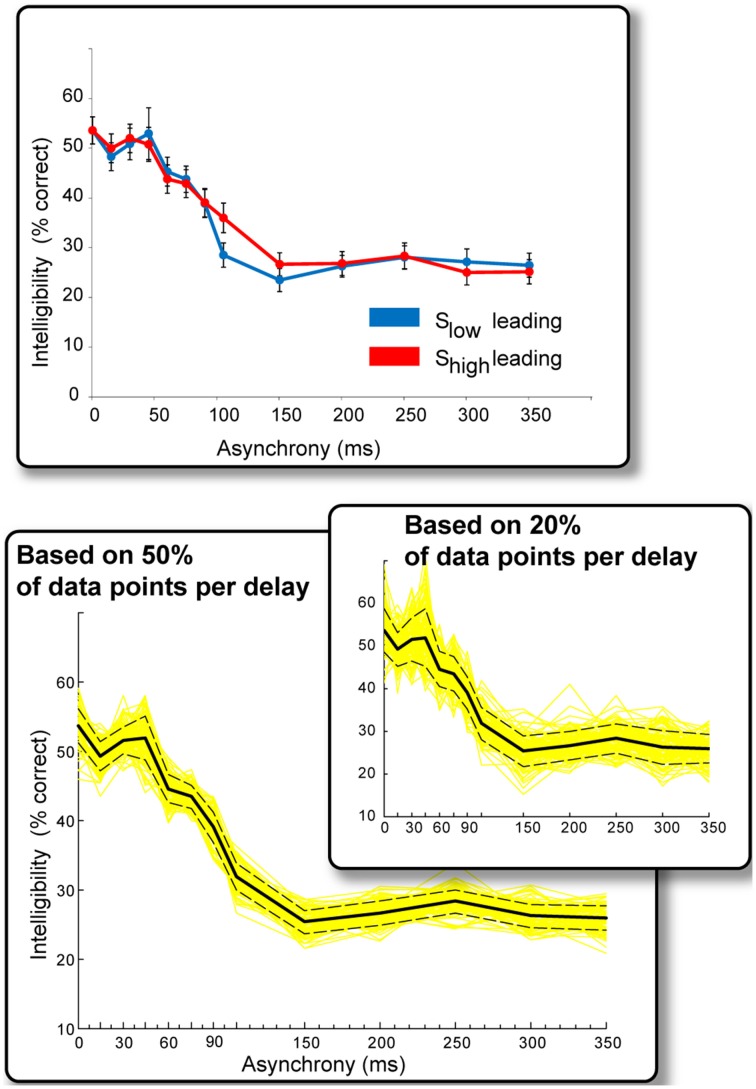
**Results of Experiment 2**. Top: Intelligibility performance as a function of onset asynchrony. The “S_low_ leading” and “S_high_ leading” conditions resulted in qualitatively similar performance and data were therefore collapsed across the two conditions in subsequent analyses. Bottom: Solid black line: Intelligibility performance as a function of onset asynchrony (collapsed over ear of presentation and S_high_/S_low_ leading). Performance can be divided into three intervals: Asynchronies <45 ms have no effect on intelligibility, performance declines sharply between 45 and 150 ms, remaining constant beyond that interval. Yellow lines reflect repeated (100 iterations) computations of the above measure using random-subsets of 50% of the data points that contributed to each delay condition. Dashed lines are the standard error of the mean derived from this procedure. The inset presents the same analysis but over 20% of data points in each delay condition. The main effects are preserved in these analyses indicating that they are a stable phenomenon.

Figure 3 summarizes the results of Experiment 2. The data were collapsed across ear of presentation because no ear effects were found. This is not surprising. As an offline study, the present experiment was not designed or optimized to test for hemispheric effects—subjects could listen to a sentence three times before providing their response and were not constrained with respect to time, making it highly unlikely that one would observe ear-specific effects.

We found no significant difference between “S_low_ leading” and “S_high_ leading” conditions (Figure 3, top), and the data associated with these conditions were also collapsed (resulting in approximately 230 data points per delay condition). The results (Figure 3, bottom) reveal several important findings. Asynchronies below ~45 ms have no appreciable effect on intelligibility (performance is roughly flat over these asynchrony values). Longer asynchronies result in a progressive decline in intelligibility until about 150 ms, at which point performance asymptotes. It is likely that S_low_ and S_high_ information cannot be bound into a usable composite representation at such large asynchronies, and subjects resort to listening to the ear that provides the most information.

We used an additional statistical procedure to ascertain the extent to which these results are stable across items and subjects. We repeated the analysis a 100 times using random subsets of 50% of the data points contributing to each delay condition (yellow lines in Figure 3). The dashed lines represent the standard error of the mean derived from this procedure. The same pattern of results is maintained even when using 20% of the data points in each delay condition (Figure 3, inset), indicating that it is indeed a stable phenomenon.

The synchronous (zero-delay) condition in Experiment 2 is equal to the BOTH condition in Experiment 1, but performance on this condition in Experiment 2 is significantly lower (54% here vs. 70% in Experiment 1). A likely explanation is that participants had much less exposure in the current experiment to the zero-delay condition. Additionally, in Experiment 1, subjects listened also to the LOW and HIGH conditions (each appeared 33% of the time), and therefore practiced attending to both sources of information. In Experiment 2, subjects heard *only* dichotic stimuli and received no relevant exposure to single modulation signals. The effect, nevertheless, is remarkable. These data invite the provocative hypothesis that in order to be combined, LOW and HIGH modulation-frequency information does not have to be extracted simultaneously. For asynchronies (in either direction) of up to ~45 ms, subjects' performance remains relatively constant, but declines sharply afterwards, suggesting a delay of ~45 ms between the extraction and the binding of these informational constituents of speech. The temporal tolerance observed here may be related to the spectral asynchronies tested in previous experiments (Greenberg and Arai, 2004).

## General discussion

The observation that the auditory system extracts information on multiple time-scales based on segregated mechanisms is attracting increasing attention (Zatorre and Belin, 2001; Boemio et al., 2005; Narayan et al.,^2006^; Giraud et al., 2007; Obleser et al., 2008; Ghitza, 2011; Giraud and Poeppel, 2012; Luo and Poeppel, 2012; Saoud et al., 2012). Imaging experiments, which have focused principally on hemispheric lateralization, support a model in which processing occurs on at least two separate time scales, 30–50 and 200–300 ms, which differentially recruit the two hemispheres. Beyond the growing body of evidence for cerebral lateralization, however, it remains unresolved what the perceptual implications of this distributed temporal processing are and how the most ecologically relevant signal, speech, incorporates such mechanisms. The stimulus employed in the present experiments allowed us to investigate certain aspects of how these distinct time scales in speech are treated separately and how they might be combined. The design of the stimuli is based on evidence (reviewed in the Introduction) for a linkage between different modulation frequencies and putative linguistic units. Filter parameters were chosen to encompass the modulation frequencies shown to be most relevant for speech: 4 Hz (~250 ms-sized temporal windows) in the LOW condition and 33 Hz (~30 ms temporal windows) in the HIGH condition. These values are further motivated by the pervasive relevance of these time ranges in non-speech and brain imaging studies (see Zatorre and Belin, 2001; Poeppel, 2003; Boemio et al., 2005; Hesling et al., 2005; Telkemeyer et al., 2009; Luo and Poeppel, 2012; Clunies-Ross et al., 2015 and references therein). Experiments 1 and 2 together suggest that when the speech signal is fractured into these two complementary HIGH and LOW parts, neither is as intelligible as the combination, and the improvement in performance is greater than would be expected from a linear combination of HIGH and LOW information, potentially reflecting a binding process that creates an acoustic, phonetic, or phonological representation that is more than the sum of its parts.

Synergistic effects have been shown to occur in the frequency domain when certain narrow spectral “slits” are combined (e.g., Warren et al., 1995, 2005). The aspects in which our findings differ from this previous work are (i) the extension to the temporal domain, as well as (ii) the *context* within which we demonstrate the effect: we show that supra-additive performance occurs for signals containing *specific* information hypothesized to be relevant for speech perception. Our data challenge common conceptions in which low-frequency modulations suffice to mediate speech recognition (e.g., Drullman et al., 1994a,b; Hermansky and Morgan, 1994; Kanedera et al., 1997). While this may be true under particular perceptual circumstances, a model that incorporates both lower and higher frequency modulations is both necessary to account for the data and is more in line with findings from psycholinguistics on the relevance of primitives of different temporal granularities (Segui et al., 1990; Decoene, 1993; Dupoux, 1993; Kakehi et al., 1996; Mehler et al., 1996; Stevens, 2002). In the present study, the choice of modulations was explicitly motivated by these independent findings from psycholinguistics and neuroimaging. Additional experiments are required to test other ranges that are not motivated by those speech considerations at stake here.

The putative “binding” of information carried in the two modulation-frequency bands apparently occurred automatically, despite subjects' conscious attempts to ignore the (reportedly “completely noisy”) high modulation-frequency signal and focus on the ear receiving the S_low_ signal. In Experiment 2 we show that listeners can withstand asynchronies as large as ~45 ms before the binding of the two information streams degrades and intelligibility deteriorates. These data suggest that S_high_ and S_low_ are likely extracted separately and in parallel from the ongoing speech signal, and provide behavioral support for accumulating brain-imaging data that show distributed processing on multiple time scales (Zatorre and Belin, 2001; Poeppel, 2003; Boemio et al., 2005; Hesling et al., 2005; Giraud et al., 2007; Ghitza, 2011; Giraud and Poeppel, 2012; Luo and Poeppel, 2012; Santoro et al., 2014).

We hypothesize that the role of the temporal envelope may be to provide segmentation (or parsing) cues: the envelope defines the relevant temporal windows from which segmental and supra-segmental information is extracted (the precise size of the segmentation window is not pre-determined but adjusted according to statistical cues in the acoustic signal). This hypothesis is consistent with MEG brain-imaging data (Ahissar et al., 2001) as well as psychophysical evidence that listeners use cues contained within the speech signal for segmentation (Huggins, 1975; Dupoux and Green, 1997; Pallier et al., 1998). The present experiments may thus be interpreted as tapping into these mechanisms by selectively eliminating segmentation cues, at least in part (the filtering was performed on the envelope despite the fine structure, carrying spectral information, remaining the same). In the LOW condition, we eliminated high-frequency modulations, and by implication, high-frequency segmentation cues. In the HIGH condition, we eliminated low-frequency modulations (low-frequency segmentation cues). Note that our signal processing is not ideal, in the sense that some low- and high-frequency modulation information remains in the signal after filtering; complete elimination of such cues is infeasible. Moreover, because the ~250 and ~30-ms intervals are average measures, the signal processing does not remove all relevant information in the same way across sentences. Nevertheless, the behavioral results are compelling and provide preliminary evidence consistent with a hypothesis, motivated by many convergent sources of evidence (Zatorre and Belin, 2001; Poeppel, 2003; Boemio et al., 2005; Hesling et al., 2005), that segmental and supra-segmental information in speech are extracted simultaneously but separately (by independent mechanisms) from the input stream from “short” (~30 ms) and “long” (~250 ms) windows of integration. These streams are then combined to generate a percept that has significant consequences for speech intelligibility.

Interestingly, these values are precisely those time periods of the theta and gamma cortical neuronal oscillations. This linking hypothesis between the time constants of speech and the time constants of neuronal oscillations has been made explicit in the literature (Poeppel, 2003; Ghitza and Greenberg, 2009; Ghitza, 2011; Giraud and Poeppel, 2012). The role of oscillations in perception and cognition is widely and energetically debated; the results we have obtained for speech and other auditory signals on balance support the hypothesis that oscillations have causal force in auditory perception and speech comprehension (e.g., Morillon et al., 2012; Doelling et al., 2014).

Building on neurophysiological studies, one line of argumentation proposes that there are two principal temporal windows operating concurrently (Poeppel, 2003; Giraud and Poeppel, 2012): one temporal window is on the order of 20–30 ms and an aspect of the cortical gamma rhythm: acoustic input is decoded with relatively high temporal resolution. A second temporal window of ~200 ms extracts acoustic information at a more global scale and is associated with the theta rhythm that parses signals into longer duration units. Such a two-timescale integration model based on oscillations also links to key time scales of visual perception (Holcombe, 2009). Indeed, the saccadic eye movements made while exploring natural scenes occur at 2–5 Hz as well, and the lower frequency, theta rhythm appears to modulate higher frequencies in a phase-dependent manner.

Although this study focused on the multi-scale nature of speech (see e.g., Rosen, 1992; Poeppel, 2003; Greenberg, 2006; Elliott and Theunissen, 2009), the mechanisms that speech processing exploits to effectively analyze the multi-time-scale constitution of the signal are likely to be of a general nature rather than speech-specific. There is abundant evidence that natural sounds of many types have such a multi-scale structure that requires analysis at multiple levels (e.g., Santoro et al., 2014). Interestingly, the evidence for this claim is most typically discussed in the context of neuroscience studies (e.g., Nelken et al., 1999; Lewicki, 2002; Singh and Theunissen, 2003; Narayan et al., 2006; Santoro et al., 2014). Using non-speech control signals that build on both temporal and spectral attributes of speech, we have also shown that such features elicit robust neuronal responses in selective regimes (e.g., Boemio et al., 2005; Luo and Poeppel, 2012; Xiang et al., 2013). In sum, the task the auditory system has to execute, namely to integrate information over different (non-overlapping) time scales in a concurrent manner, has been well-documented in the neuroscience literature.

In contrast, the investigation of this type of parallel processing using purely psychophysical paradigms has not been widely reported. Many experiments address the question of temporal integration in hearing and vision, but typically not in a multi-scale way. Most studies aim to identify “the” integration constant and derive other phenomena from a single, monolithic integration value. Because some studies find short time constants (for example for modulation detection; Viemeister, 1979) and some studies point to much longer time constants (for example for loudness integration; Fletcher, 1933), the conflicting data have been argued to point toward an integration–resolution paradox (De Boer, 1985; Green, 1985). The notion that multiple streams of input signal are being analyzed concurrently, on different scales, is not widely tested in the behavioral literature. To our knowledge, this is one of the first studies to use purely behavioral measures to assess the contribution of information on multiple time scales, at least for speech.

### Conflict of interest statement

The authors declare that the research was conducted in the absence of any commercial or financial relationships that could be construed as a potential conflict of interest.
